# Susceptibility-Weighted Imaging Manifestations in the Brain of Wilson’s Disease Patients

**DOI:** 10.1371/journal.pone.0125100

**Published:** 2015-04-27

**Authors:** Jinjing Yang, Xiaohu Li, Renmin Yang, Xuen Yu, Changliang Yu, Yinfeng Qian, Yongqiang Yu

**Affiliations:** 1 Department of Radiology, The First Affiliated Hospital of Anhui Medical University, Hefei, China; 2 Department of Neurology, The Hospital Affiliated of Anhui College of TCM, Hefei, China; Fatebenefratelli Foundation for Health Research and Education, ITALY

## Abstract

**Purpose:**

It is well known that patients with Wilson’s disease (WD) suffer copper metabolism disorder. However, recent studies point to an additional iron metabolism disorder in WD patients. The purpose of our study was to examine susceptibility-weighted imaging (SWI) manifestations of WD in the brains of WD patients.

**Methods:**

A total of 33 patients with WD and 18 normal controls underwent conventional MRI (Magnetic resonance imaging) and SWI. The phase values were measured on SWI-filtered phase images of the bilateral head of the caudate nuclei, globus pallidus, putamen, thalamus, substantia nigra, and red nucleus. Student’s *t*-tests were used to compare the phase values between WD groups and normal controls.

**Results:**

The mean phase values for the bilateral head of the caudate nuclei, globus pallidus, putamen, thalamus, substantia nigra, and red nucleus were significantly lower than those in the control group (P < 0.001), and bilateral putamen was most strongly affected.

**Conclusions:**

There is paramagnetic mineralization deposition in brain gray nuclei of WD patients and SWI is an effective method to evaluate these structures.

## Introduction

Wilson's disease (WD) is an autosomal recessive inherited copper metabolism disorder. Mutations in the ATP7B gene disturb copper transport, excretion into the bile, and incorporation of copper into apo-ceruloplasmin. These disorders lead to pathological copper accumulation in many tissues, especially in the liver and brain. WD is a rare treatable inherited disease [1]. Long-term treatment with anti-copper agents such as penicillamine and zinc is the standard therapy; however, some patients respond poorly and the accumulation of other minerals in addition to copper must also be considered.

WD lesions on brain MRI (Magnetic resonance imaging) generally present as areas of symmetric hypointensity on T1-weighted images and as hyperintense areas on T2-weighted images, due to neuronal edema, necrosis, cavernous transformation or gliosis. However, some studies have also found hypointense lesions on T2-weighted images in WD patients and hypothesized a paramagnetic effect of metal ion accumulation, such as copper and iron [2, 3]. Hitoshi et al. [2] suggested that iron played a more important role than copper in reducing signal intensity on T2-weighted images because the lesions in WD patients under long-term copper chelation therapy appeared similar to those in untreated patients. In addition, hypointense signals on T2-weighted images in WD are similar to signals resulting from iron deposition in patients with Parkinson's disease (PD), Alzheimer’s disease (AD) and multiple sclerosis (MS). Recently, a new MRI technique, susceptibility-weighted imaging (SWI), combines phase and magnitude images and has been shown to be very sensitive in the detection of iron accumulation [4, 5], enabling measurement of iron in tissue as low as 1 μg/g *in vivo* [6, 7]. In our study, we measured phase values of bilateral gray matter nuclei on SWI-filtered phase images, and observed SWI manifestations in the brains of WD patients.

## Materials and Methods

### Subjects

This study was approved by the Institutional Review Board at the First Affiliated Hospital of Anhui Medical University (Hefei, China) and all study participants provided written, witnessed, informed consent. Consent from next of kin, caretakers, or guardians provided signed informed consent for minors. This research was conducted in accordance with the Declaration of Helsinki.

A total of 33 patients with WD were enrolled from January 2011 to May 2013. There were 17 females and 16 males, ranging in age from 9 to 40 years with mean age of 21.18±7.35 years. Diagnoses were based on clinical symptoms, abnormal copper metabolism (decreased level of serum ceruloplasmin < 20 mg/dl and increased 24 h urine copper excretion > 100 μg/24 h), and presence of the Kayser–Fleischer ring. For controls, 18 age- and sex-matched healthy individuals were selected, free from any neurological or medical conditions, including eight females and ten males with a mean age of 23.78±1.96 years.

### MRI data acquisition

All studies utilizing both conventional MRI and SWI were performed with a 3.0T scanner(GE Signa HDx, General Electric, Milwaukee, WI, USA.) equipped with a standard eight-channel head coil. Conventional MRI were obtained with the following parameters: (1) Axial T1-weighted images: TR/TE/NEX, 2000 ms/20 ms/1.00; FOV, 22 cm × 17.6 cm; section thickness, 5 mm with a 1.5 mm gap; matrix size, 320 × 224; (2) Axial T2-weighted images: TR/TE/NEX, 4400 ms/120 ms/1.00; FOV, 22 cm × 17.6 cm; section thickness, 5 mm with a 1.5 mm gap; matrix size, 384 × 256; (3) Fluid-attenuated inversion recovery (FLAIR): TR/TE/TI/NEX, 9000 ms/150 ms/2250 ms/1.00; FOV, 22 cm × 22 cm; section thickness, 5 mm with a 1.5 mm gap; matrix size, 320×192. The SWI parameters were as follows: TR = 55 ms; TE = 5.5–50.4 ms; flip angle = 20°; FOV, 22 cm × 18 cm; section thickness, 2 mm with no gap; matrix size, 416 × 256.

**Fig 1:**
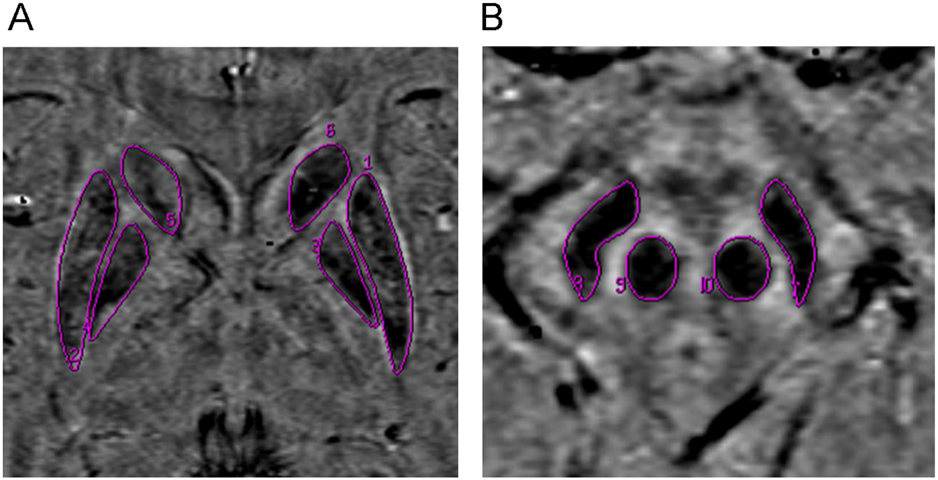
ROIs used for calculation of mean phase values. Phase images show the ROIs used for the measurement of the mean phase values in bilateral PUT (1, 2), GP (3, 4), HCN (5, 6), (A) SN (7, 8), and RN (9, 10) (B).

All SWI images were post-processed using our in-house Functool software on the workstation ADW4.4. Following post-processing, high-pass filtered phase and magnitude images were automatically presented. Regions of interest (ROIs) were manually traced on the SWI-filtered phase images, including the bilateral head of caudate nuclei (HCN), globus pallidus (GP), putamen (PUT), thalamus (THA), substantia nigra (SN), and red nucleus (RN), as shown in Fig 1. The largest area of each nucleus was selected for all study participants. To ensure data accuracy, all ROI structures were measured three times and mean phase values were calculated.

### Data analysis

All statistical computations were conducted using the IBM SPSS 19.0 statistical package (Armonk, NY, USA). The mean and standard deviation were calculated for statistical analysis. Two-sample *t*-tests were used to determine if there was a significant difference between the mean phase values of gray matter nuclei in WD groups and normal controls. P values < 0.05 were considered statistically significant.

## Results

**Fig 2:**
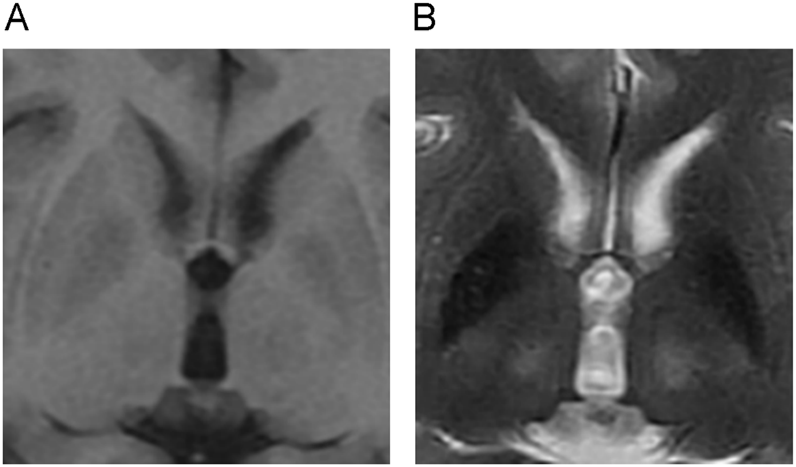
Conventional MRI phase images of WD patients. Many patients showed hypointense areas on T1-weighted images(A) and hyperintense areas on T2-weighted images(B).

**Fig 3:**
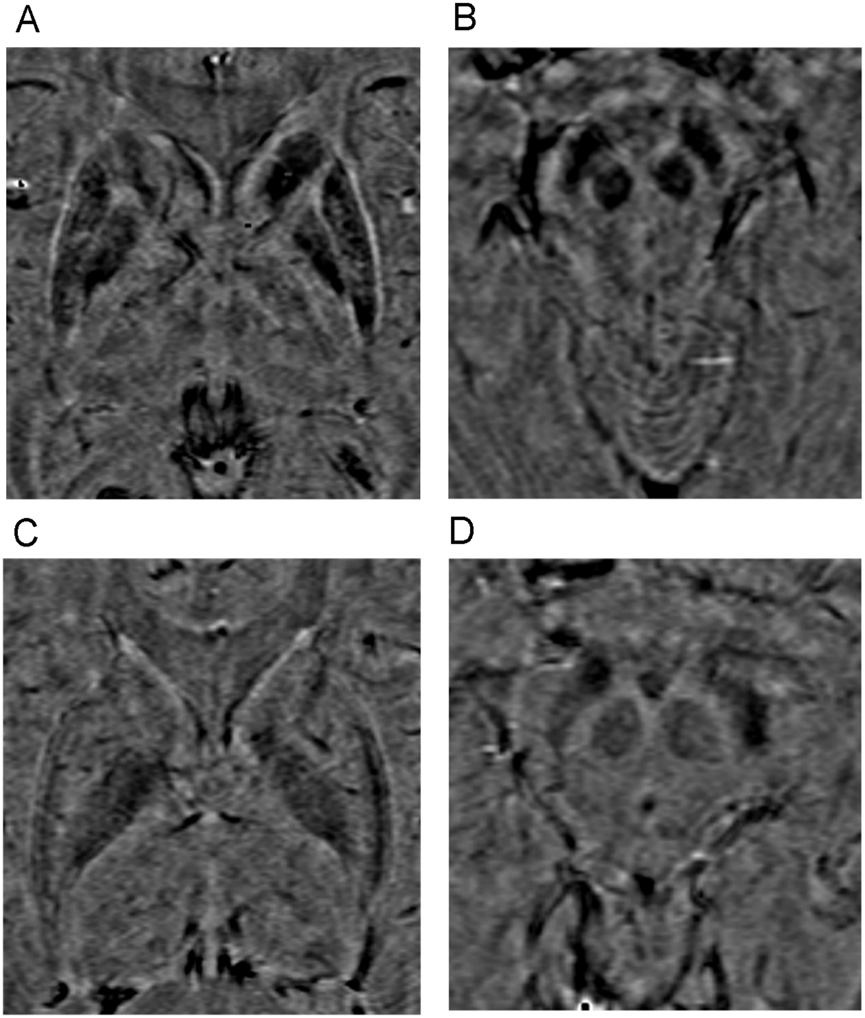
Phase images of WD groups and sex- and age-matched healthy controls. A, B: WD patients. C, D: Control patients. The signal intensity on phase images of gray matter nuclei in WD groups (A, B) was lower, and the outlines of gray matter nuclei were clearer than in control groups (C, D).

All images of WD patients and healthy controls were judged acceptable for analysis. Typical abnormalities of WD, including hypointense signals on T1-weighted images and hyperintense signals on T2-weighted images, were present on conventional MRI of many patients (Fig 2A and 2B). HCN, GP, PUT, THA, SN, and RN clearly presented as hypointense on SWI-filtered phase images. Compared to healthy controls, the signal intensity on phase images of gray matter nuclei in WD groups was greatly reduced and the outlines of gray matter nuclei were clearer (Fig 3A–3D). In WD groups, the low intensity signals of bilateral HCN, GP, and PUT were unevenly distributed, with the edges of those nuclei showing the lowest intensities.

**Fig 4:**
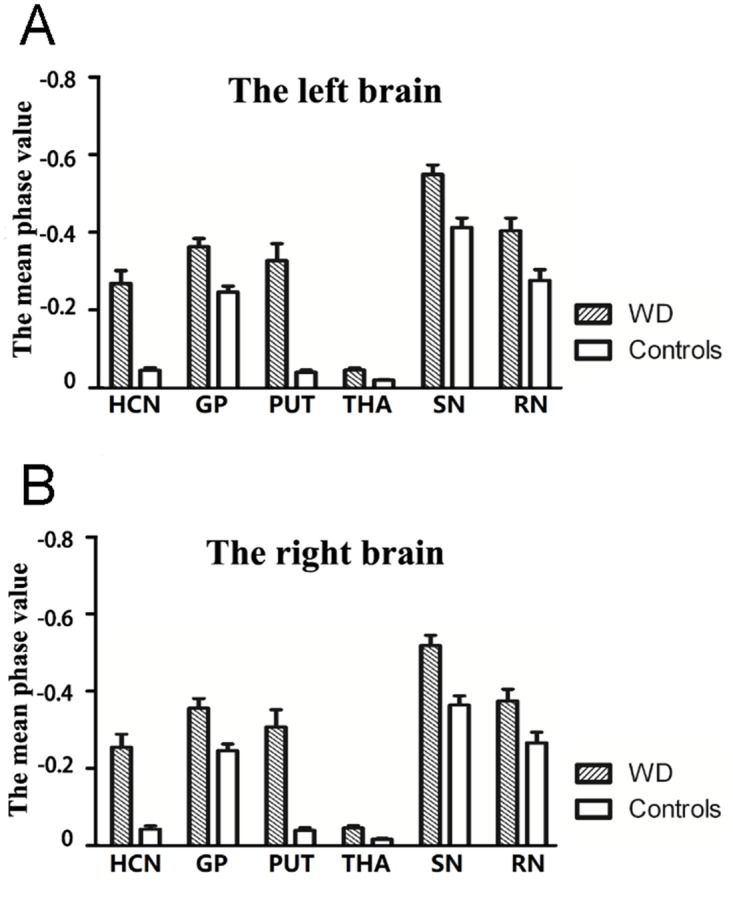
Plots of mean phase values in the left and right brains of WD and control groups. The plots show the mean phase value of bilateral gray matter nuclei in left (A) and right (B) brain of WD and control groups. The differences in phase values for all nuclei on both sides of the brain were significantly different between WD groups and normal controls (P<0.001), with the most substantial difference occurring in PUT.

There were no significant age and sex differences between the WD groups and healthy controls, and no differences between the left and right gray matter nuclei. The phase values for bilateral HCN, GP, PUT, THA, SN, and RN in WD groups were markedly reduced and were significantly different compared to normal controls (P < 0.005; Fig 4A and 4B). The difference in signal intensity between WD patients and healthy controls was largest in bilateral PUT.

## Discussion

It is well known from clinical biochemical data that patients with WD suffer copper metabolism disorder, and an increasing number of studies now suggest that iron metabolism is disturbed as well. It has been determined that copper and iron metabolism are closely linked [8]. As a consequence of the mutated ATP7B gene in WD patients, not only is ceruloplasmin decreased, but the incorporation of copper into enzymes is also impaired [9]. Copper-dependent enzymes play an important role in iron metabolism. Ferric iron is reduced to ferrous iron by the copper-dependent reductase duodenal cytochrome B (Dcytb) before it is taken up by enterocytes [10]. Iron export via ferroportin is mediated by hephaestin, a membrane-bound copper-dependent ferroxidase [11]. In addition, upregulation of iron-dependent enzymes partially compensates for the disturbance of respiratory enzymes in WD (by a metabolic copper disorder) and leads to higher cellular demand for iron [9].

Hypoceruloplasminemia is a major risk factor for iron accumulation in WD, and evidence supports a critical role for ceruloplasmin in iron metabolism. Ceruloplasmin is an enzyme with highly efficient ferroxidase activity that catalyzes the conversion of Fe2^+^ into Fe3^+^ and is essential for iron transport across cell membranes [12]. Ceruloplasmin also facilitates cellular iron uptake [13] and the release of iron from HepG2 human liver cells [14]. In a study of WD gene knockout mice [10], serum iron, serum transferrin saturation, and blood hemoglobin levels were significantly lower in Atp7b (-/-) mice compared with controls. The knockout mice also displayed slightly elevated hepatic iron content, which indicated that decreased serum iron parameters were most likely related to low serum ceruloplasmin oxidase activity and were not the result of total body iron deficiency.

The subcellular co-existence of copper with iron has been observed in pretreatment liver biopsy specimens from WD patients. Following long-term copper chelation therapy, copper content was significantly reduced and iron content elevated, and in almost all hepatocyte lysosomes, copper was replaced by iron [15]. A ^52^Fe-citrate PET study found that cerebral iron uptake was significantly higher in WD patients compared to healthy volunteers [9]. SWI, a novel MRI technique, takes advantage of the magnetic susceptibility differences of various tissues. These differences can be differentiated in high-pass filtered SWI phase images [16]. SWI has significant advantages over conventional MRI sequences, with a fully velocity-compensated three-dimensional sequence, high spatial resolution and signal-to-noise ratio, and enhanced susceptibility sensitivity. Iron is a more paramagnetic element that changes the local magnetic field in the presence of an externally applied magnetic field and leads to a change in phase value that presents as reduced signal intensity on filtered phase images [17].

The utility of SWI in evaluating iron deposition has been repeatedly confirmed. For example, SWI had been used to estimate brain iron content in patients with PD and MS [18, 19]. In these studies, hypointensity on filtered phase images was consistent with pathological iron deposition regions, and the phase values were closely correlated with disease duration and clinical scores, making them useful as *in vivo* biomarkers to objectively evaluate disease status. Furthermore, SWI has also been used to investigate age-related iron deposition [20], splenic siderotic lesions [21] and hepatocellular carcinoma [22].

In our study, HCN, GP, PUT, THA, SN, and RN in the WD group showed marked hypointensity on SWI and the phase values in these areas were significantly lower than in the control group (P < 0.005). From these results, we can conclude there was paramagnetic mineral deposition in the gray nuclei of WD patients. If copper is paramagnetic, the observed abnormal signal on SWI may reflect copper deposition. However, the contribution of copper to the degree of susceptibility effects observed on SWI has not been determined. In a study by Lee et al. [23], bilateral SN, RN, lentiform nucleus and caudate nucleus showed dark paramagnetic signals on SWI, and no evidence of calcification or hemorrhage was observed on brain CT. After adequate de-coppering treatment with Trientine, neurological symptoms deteriorated in the same patient and aggravated lesions were shown on follow-up T2-weighted images; however, paramagnetic signals in basal ganglia on SWI were unchanged. In another study by Lee et al. [24], abnormal paramagnetic signals on SWI were observed not only in *de novo* patients with early stage WD, but also in patients undergoing a long-term copper chelating therapy. This may be due to inflammation induced by copper damage, with heavy influx of iron-laden macrophages and other inflammatory cells. Histopathological studies found hemosiderin-laden macrophages in the PUT and phagocytes containing iron pigment in the GP and SN [25]. Therefore, we believe that the abnormal paramagnetic signals on SWI in WD patients were mainly caused by iron deposition.

The brain requires a ready supply of iron for normal neurological function. Ferritin is the most common form of non-heme iron in brain. Iron content in the extrapyramidal system is high, while in the cortex it is lower, and white matter is almost free from iron. The abnormal hypointensity on filtered phase images in our study were mostly located in gray matter nuclei, such as GP, PUT, SN, and RN, similar to regions of iron deposition (hypointense regions) observed on SWI in PD and to sites of abnormal paramagnetic signals on SWI in WD patients reported by Lee et al. [23].

The PUT is the most frequently involved site in the brain of WD patients [26, 27]. In our study, we observed that the difference between WD groups and healthy controls was most prominent in bilateral PUT and that signal loss was greatest at the edge of the PUT.

Comparing conventional MRI to SWI, we found that the typical abnormalities observed on conventional MRI are useful for diagnosing WD, but MRI cannot determine if there is paramagnetic mineralization, which is an advantage of using SWI. A few reports have suggested that single photon emission computed tomography (SPECT) may also be useful for diagnosis of WD [28].

The main limitation of this study was the lack of an SWI comparative study between pre- and post-treatment WD patients. In addition, the clinical data available on our WD patients were not comprehensive. In a future study, we would like to collect more clinical data and analyze the correlation between clinical data and image features. The exact nature of hypointense lesions observed in WD patients is still obscure and further SWI pathological studies are needed to illuminate their origin.

In conclusion, SWI is a relatively safe, simple and noninvasive examination procedure *in vitro* which can be used to assess brain paramagnetic mineral deposition in WD patients.
